# Paralysis of the Intestines

**Published:** 1845-05

**Authors:** 


MEDICAL SOCIETY OF LONDON, MARCH 10, 1845.

*Paralysis of the Intestines.—Obstinate constipation of the Bowels.* Dr. Waller related the case of a gentleman exceedingly corpulent, and forty-six years of age, the subject, for a long period, of very large irreducible hernia—possibly, indeed, congenital. He had been the subject of constipation on several occasions, attended with great pain in the hernia, which symptoms, however, usually gave way under the employment of rest and mild aperients. He was in the habit of taking "antibilious pills," and on the morning of the 27th of November last, he took two of these, was freely purged on the 28th, and on the 29th pursued his usual avocation as a shopkeeper; but in the afternoon of that day was seized with a violent, apparently spasmodic, pain in the stomach and abdomen. There were intermissions of pain, but great inconvenience from tympanitic distention of the stomach. He was free from fever. Calomel and opium, followed by a saline purgative, were administered, but without relief. An enema, and subsequently castor oil, were given, with the effect of bringing away a few scybalæ, but otherwise without benefit. He was afflicted with frequent vomiting, the matters ejected being the fluids swallowed, and thick ropy mucus. The hernia was examined, and was without pain, and less, instead of larger, as in previous attacks. Various purgatives were given without benefit, but a terebinthinate injection was returned with much force, and accompanied with flatus, but no feculent discharge followed. On the next day, the 30th, he was in the same condition; there was, however, some uneasiness of the abdomen, not amounting to pain. He was bled to eight ounces, as a matter of precaution, and afterwards placed in a warm bath. He remained the same on the 1st of December, and Mr. Solly was requested to see him. This gentleman agreed in opinion with Dr. Waller, and considered that, although the *general* symptoms were those of strangulation, yet the local condition of the hernia did not warrant an operation. A few leeches were applied over the hernia, and calomel and opium given internally, and also an injection of turpentine, with the addition of tincture of assafœtida. No relief followed, and the next day twenty leeches were applied to the abdomen. The matter vomited in the fore part of this day on one occasion appeared stercoraceous. In the evening vomiting ceased, and a drastic purgative of scammony, calomel, and gamboge, was administered. No relief followed, and the feeling of distention became most distressing; the operation of opening the hernial sac was determined upon, and Mr. Solly performed the operation.

On opening the sac, a large piece of omentum, loaded with fat, protruded itself; behind this there was a large portion of colon, of healthy appearance, slightly darker than another portion subsequently discovered, which was situated behind it, but neither presenting any appearance of strangulation. The omentum was adherent in many parts, and greatly thickened, so that it was found necessary to remove a portion, which weighed twelve ounces and a half. After much difficulty the gut was returned, Mr. Solly having previously divided a few fibres of the abdominal ring. No portion of the exposed intestine appeared to have been injured, and, in fact, no stricture was discovered; the wound was then dressed in the usual manner.

For a short time afterwards the patient appeared a little easier, but, on watching him through the day, it was evident he was no better. As there were no symptoms of inflammation, however, it was still thought there was a chance of his recovering. Whilst consulting on the propriety of administering a drastic purgative, they were summoned to his bedside, and ascertained that a very slight evacuation had taken place. This, however produced no relief; he gradually got worse, and symptoms of rapid sinking manifested themselves; vomiting of a large quantity of dark-coloured fluid became incessant; distention increased, and at half past seven A. M., 4th December, he died, a small fluid motion having passed an hour and a half previously.

On examination of the body after death, no traces of disease manifested themselves. The hernial sac was perfectly empty, and there was no appearance of peritoneal inflammation. The whole of the small intestines, cœcum, and the greater portion of the transverse arch of the colon, were enormously distended with flatus, a portion of the latter (transverse arch) was not so distended, and this was the part first discovered on opening the sac. It was scarcely smaller than natural, but was less than the distended portion above, but neither on its peritoneal nor mucous surface did it present the slightest appearance of having been strictured; it was adherent to the peritoneum, close to the mouth of the sac. Perhaps the diminution of its calibre was occasioned by the hardened omentum lying upon it in the sac. The other portion of the colon which had been in the sac was the sigmoid flexure, and this is rather larger than the last-named portion. There was a very large accumulation of feculent matter both in the large and small intestines, but it was perfectly liquid, and there was no scybalæ.—*London Lancet.*

